# Technical Adaptations in Kyphoplasty for T5 Vertebral Collapse With Sharp Kyphotic Angulation

**DOI:** 10.7759/cureus.92715

**Published:** 2025-09-19

**Authors:** Justin Nguyen, Steven M Sasser, Navneet Sharma, Matthew D Overturf

**Affiliations:** 1 Medical School, Edward Via College of Osteopathic Medicine, Monroe, USA; 2 Physical Medicine and Rehabilitation, Edward Via College of Osteopathic Medicine, Monroe, USA; 3 Physical Medicine and Rehabilitation, Green Clinic, Ruston, USA; 4 Anatomical Sciences, Edward Via College of Osteopathic Medicine, Monroe, USA

**Keywords:** curved needle, gibbus deformity, kyphoplasty, orthopedics, osteoporosis, physical medicine and rehabilitation, vertebral compression fracture

## Abstract

Vertebral compression fractures (VCFs) are a common injury pattern observed in elderly individuals with osteoporosis. However, the presence of significant spinal deformity and/or advanced vertebral collapse can render kyphoplasty technically challenging. We present the case of a 65-year-old woman suffering from severe osteoporosis who experienced a T5 compression fracture attributed to a pronounced thoracic kyphosis and osteoporosis, with approximately 75% reduction in vertebral height. Despite initial conservative management, the patient's pain persisted. During the procedure, the concave geometry of the collapsed vertebrae posed considerable access challenges, necessitating a transition from the originally planned straight-needle trajectory to a curved-needle approach. This adaptation facilitated safe navigation through the altered anatomy, enabling balloon deployment and cement augmentation. The patient reported complete cessation of pain postoperatively, with continued relief observed at follow-up. This case highlights the importance of curved instrumentation in overcoming complex anatomical constraints, thereby minimizing procedural risks and enhancing outcomes in intricate osteoporotic VCFs.

## Introduction

Vertebral compression fractures (VCFs) are a prevalent form of injury among elderly populations, with approximately 1.5 million cases in the broader United States population [1]. A loss of 15%-20% in vertebral height defines a VCF [2]. Epidemiological studies show a marked rise in vertebral fracture incidence with age, affecting both sexes but occurring more often in women due to lower bone mineral density. Population-based data indicate that prevalence increases from about 3% in individuals under 60 to nearly 20% in those 70 and older, underscoring age as a major risk factor [1,3,4]. These fractures can significantly affect patients' quality of life and are often caused by persistent axial loading and pressure on the vertebrae [5].

Kyphoplasty is regarded as a definitive treatment for osteoporotic compression fractures [6]. Notably, balloon kyphoplasty has demonstrated effectiveness as an alternative treatment for osteoporotic compression fractures in patients suffering from severe pain or pain refractory to conservative management [6]. Moreover, this minimally invasive procedure employs a percutaneous technique to introduce an inflatable balloon and inject polymethylmethacrylate, a bone cement used to stabilize vertebral body fractures [6,7]. This technique ultimately stabilizes the vertebral fracture, restores mechanical function, and alleviates pain [7]. Although kyphoplasty is a well-established intervention for painful osteoporotic compression fractures, its technical limitations and complexity become apparent in cases involving severe vertebral collapse or fixed spinal deformities [8]. Literature addressing modifications to overcome these challenges, particularly in the thoracic spine, remains sparse. This situation may also prompt reconsideration and potential redesign of kyphoplasty procedures to enhance access in anatomically challenging cases, thereby reducing risk and optimizing patient outcomes.

We present a case involving a 65-year-old woman with a severe T5 vertebral collapse leading to a severe and sharp thoracic angulation, where standard instrumentation posed significant clinical challenges. This report describes a technically complex case of T5 vertebral collapse with fixed thoracic kyphosis, where conventional straight-needle kyphoplasty was not feasible and curved instrumentation enabled successful treatment.

## Case presentation

**Figure 1:**
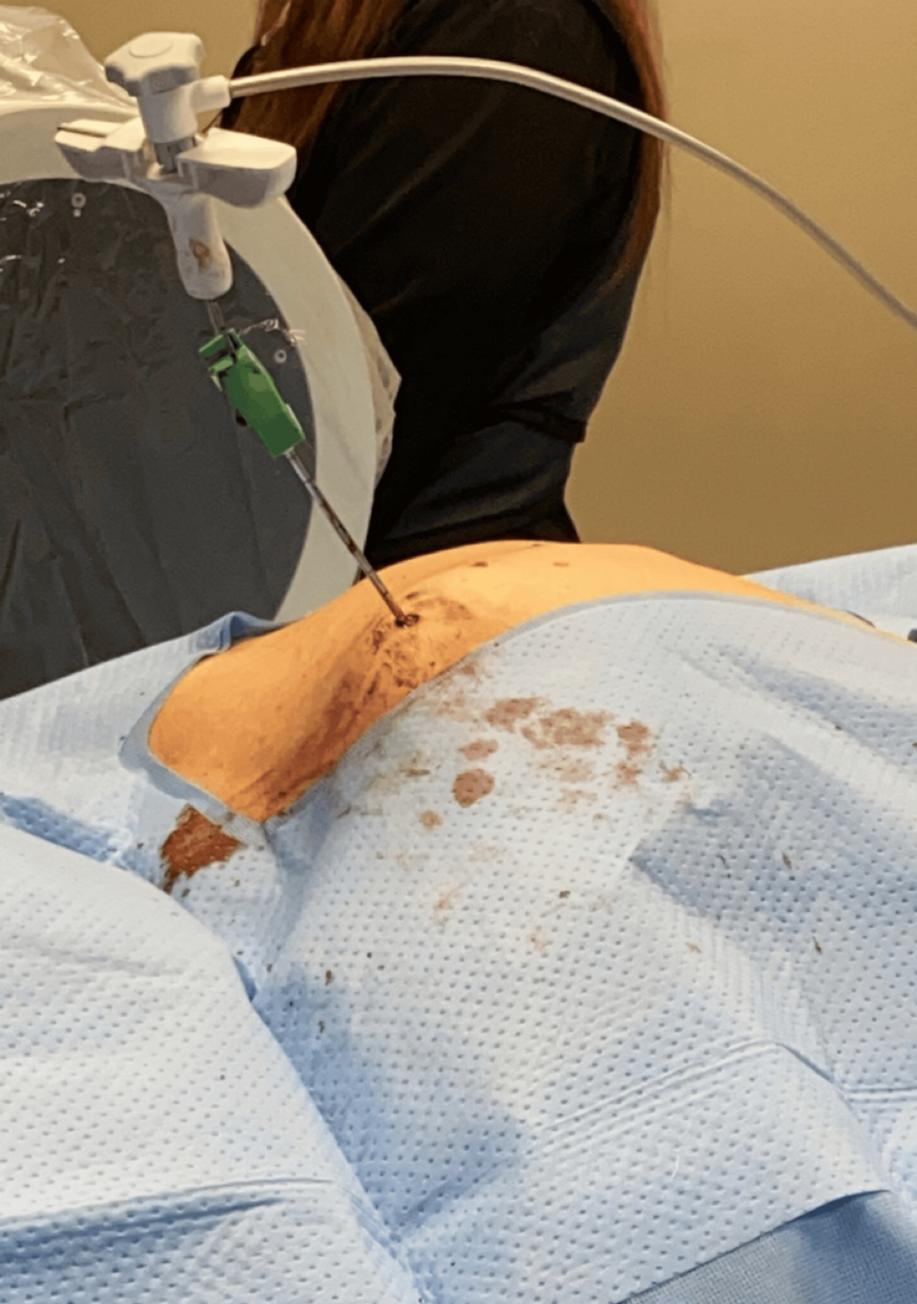
Image of the patient with marked thoracic kyphosis taken during procedure

A 65-year-old Caucasian woman presented to our interventional pain clinic for the evaluation of isolated mid-thoracic back pain. The onset of the pain was sudden and began six months prior to her initial consultation, persisting since then. The patient described the pain as a constant dull, aching sensation exacerbated by activity. She denies any trauma preceding the onset of the pain. Furthermore, she denies any fecal or bladder incontinence, radiation of pain, or extremity numbness/weakness. She reports that her pain averages 8/10 on her pain scale and is the only limiting factor to her motor strength. When asked about limitations, the patient stated that the pain affects her ability to perform activities of daily living, such as getting dressed, lifting cookware, and twisting while cleaning her house. She also endorses decreased sleep due to the inability to find a painless position of ease. Even with the addition of opioids and a thoracic-lumbar-sacral orthosis for pain control and stability, she reports that the pain is immense and limits her physical ability, including walking. Her medical history includes osteoporosis and hypertension. Her medication regimen consists of alendronate, carvedilol, hydrocodone, methocarbamol, naproxen, and valsartan. The patient has a history of tobacco use but denies alcohol consumption and the use of other illicit drugs. On physical examination, inspection revealed no swelling or erythema of the mid-back; however, marked thoracic kyphosis was observed (Figure 1).

**Figure 2:**
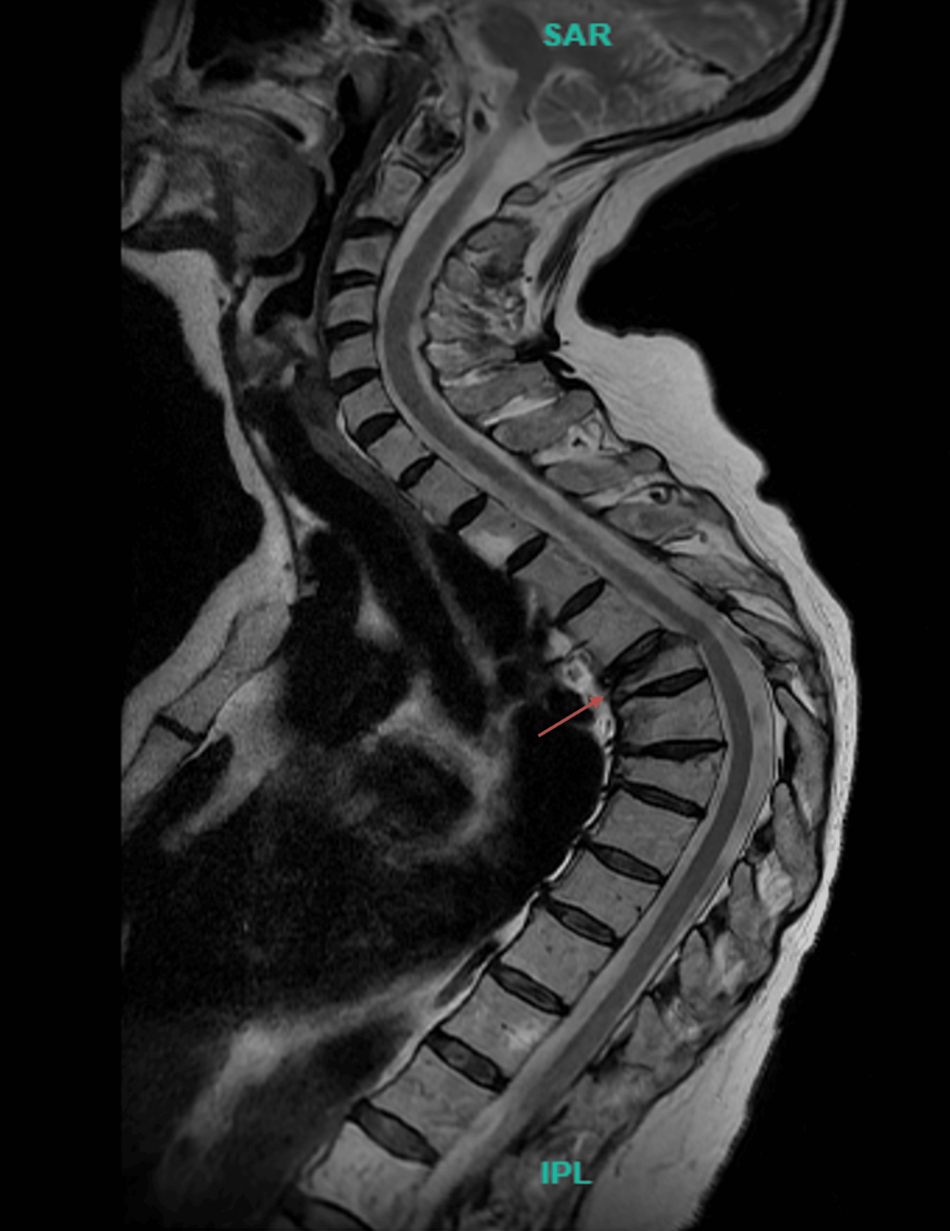
MRI indicating a compression deformity at the T5 vertebral body level, with a 75% reduction in height (red arrow)

The mid-thoracic region was tender to palpation without radiation. Upper and lower extremity strength, sensation, and reflexes were intact. Movement in all planes reproduced her pain. Consequently, a chest radiograph and MRI were ordered. The chest radiograph demonstrated a compression fracture of the T5 vertebra with approximately 75% height loss. The T6 vertebra exhibited a 25% height loss, and T7 showed approximately 50% height loss. Anterior wedging was noted at all three levels. A significant kyphotic curve with the apex at T6 was present. No significant spondylolisthesis was observed on imaging. The MRI (Figure 2) revealed an acute or subacute compression deformity of the T5 vertebral body with 75% height loss and associated bone edema, without retropulsion.

Additionally, chronic compression deformities involving the superior endplates of T6 (25% height loss) and T7 (50% height loss) were identified, lacking evidence of retropulsion or associated bone marrow edema. Mild, diffuse multilevel thoracic spondylosis with disc desiccation was also observed. No focal disc herniation, spinal canal stenosis, or neural foraminal narrowing was detected.

The working diagnosis attributed the VCF to her underlying osteoporosis and marked kyphosis. Kyphoplasty was deemed the most appropriate treatment; however, the patient's insurance denied authorization, recommending conservative modalities such as pain medication and physical therapy. Pain medication and physical therapy were prescribed for four weeks, with a follow-up scheduled to monitor progression. At the four-week follow-up, the patient stated she was only able to participate in one therapy session due to worsening pain, and the pain medication did not provide adequate pain control, with the average being an 8/10 over the four weeks. Since pain medication proved inadequate and physical therapy exacerbated her condition, resulting in a pain intensity of 10/10, insurance subsequently approved and scheduled kyphoplasty via fluoroscopy.

**Figure 3:**
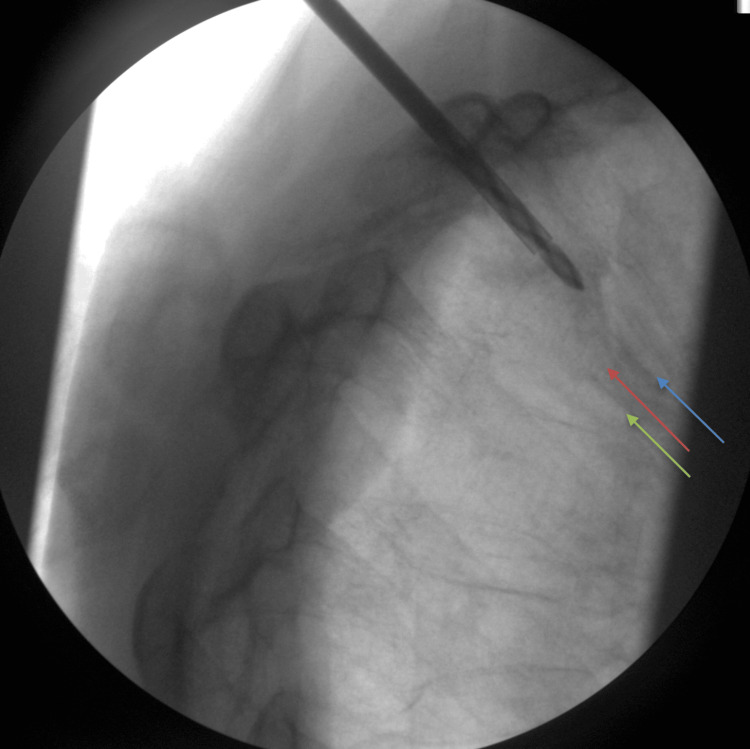
Intraoperative fluoroscopic imaging, illustrating a straight needle entering the vertebral body via the pedicle. Note how the straight needle is in the posterior portion of the vertebral body and is unable to access the affected area for cement deployment, making delivery of cement unsafe for the patient. The central portion of the T5 vertebral body depicting the fracture is indicated by the red arrow. Superior endplate of the T5 vertebrae is indicated by the blue arrow. The inferior endplate of the T5 vertebrae is indicated by the green arrow

During the procedure, intraoperative challenges were encountered. The thoracic kyphosis observed (Figures 1, 2) had to be considered to ensure safe and accurate navigation of the altered anatomical landmarks. In some modalities, trocars are introduced bilaterally through the posterior pedicle to access the fractured space safely. Subsequently, a needle is inserted to deploy a balloon aimed at restoring bone height, followed by injection of bone cement. Due to the fracture presentation in Figure 3, there was insufficient space to advance the straight needle for balloon deployment.

**Figure 4:**
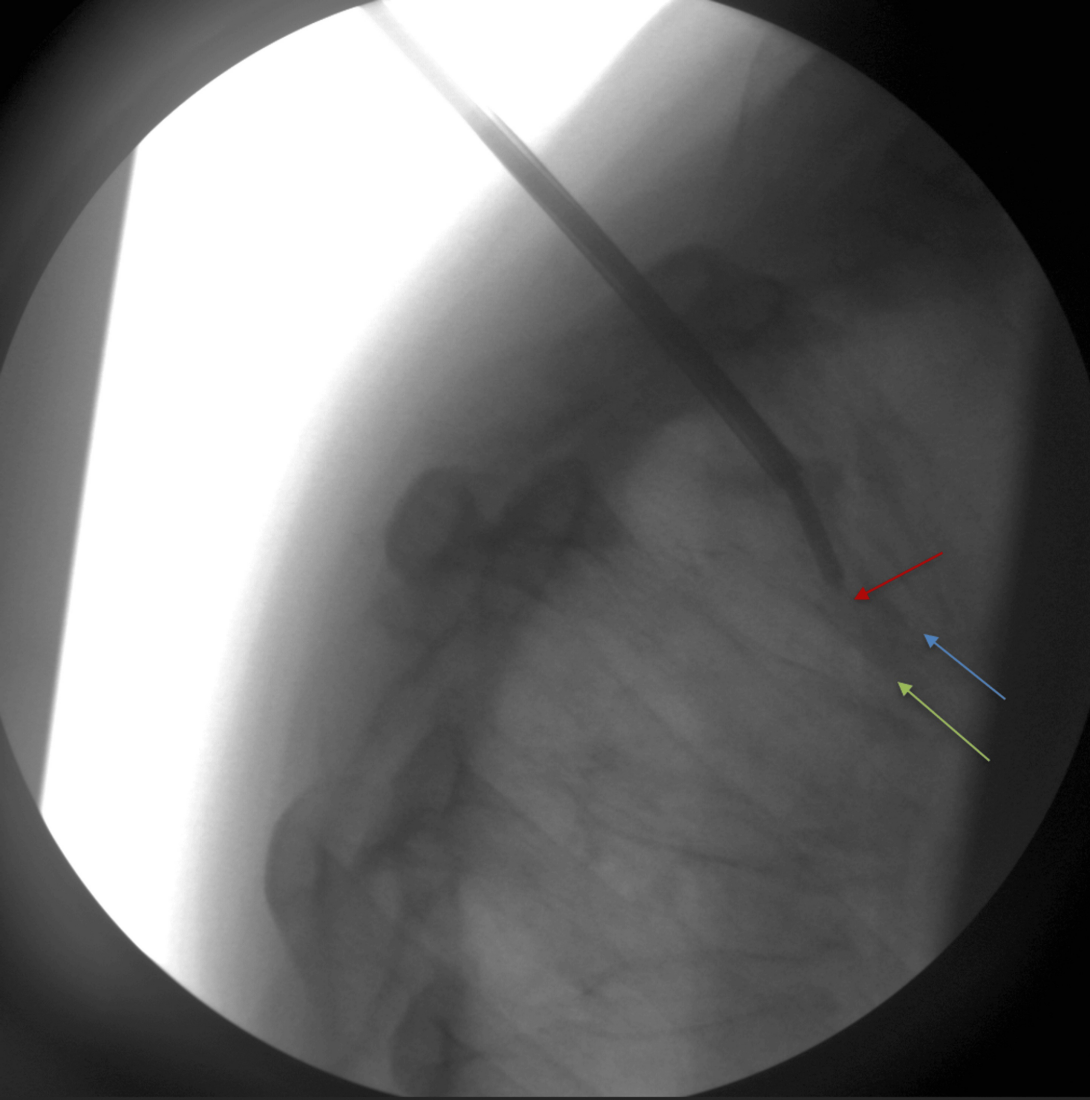
A curved needle was substituted due to difficulty accessing the fractured area with a straight needle. This facilitated access to the central portion of the T5 vertebral body containing the fractured region for the deployment of the balloon and cement (red arrow). Accessing the central area allows for the safest delivery of cement while maximizing the opportunity to regain vertebral height. Superior and inferior endplates of the T5 vertebrae are indicated by the blue and green arrows, respectively

As shown in Figure 4, the trocar angle did not permit the safe placement of the straight needle.

**Figure 5:**
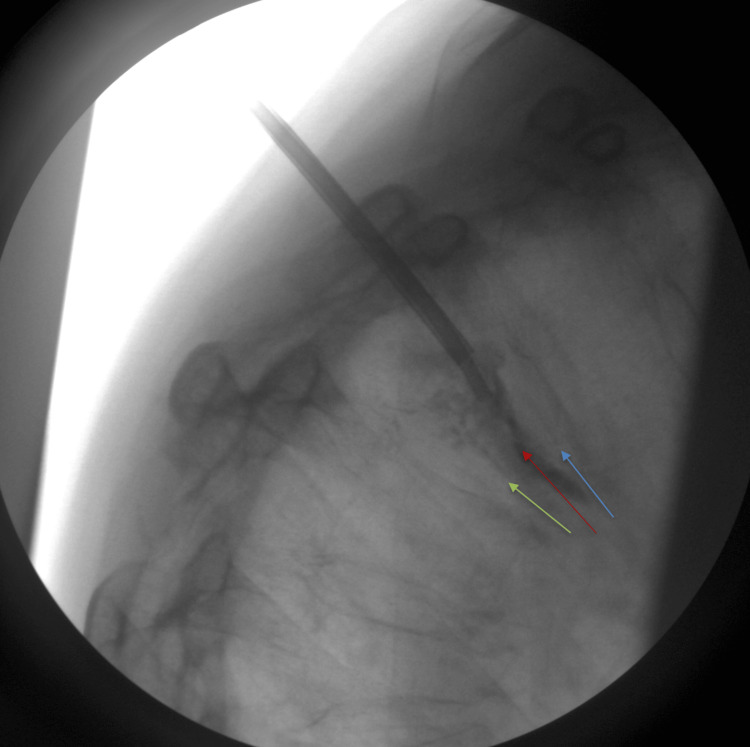
A balloon was deployed to restore bone height, followed by the introduction of bone cement (PMMA) into the space to ensure fracture stabilization (red arrow). Superior and inferior endplates of the T5 vertebrae are indicated by the blue and green arrows, respectively PMMA: polymethylmethacrylate

To address this, a curved needle was introduced to navigate beneath and around the deformity, adequately covering the entire vertebral body (Figure 5).

**Figure 6:**
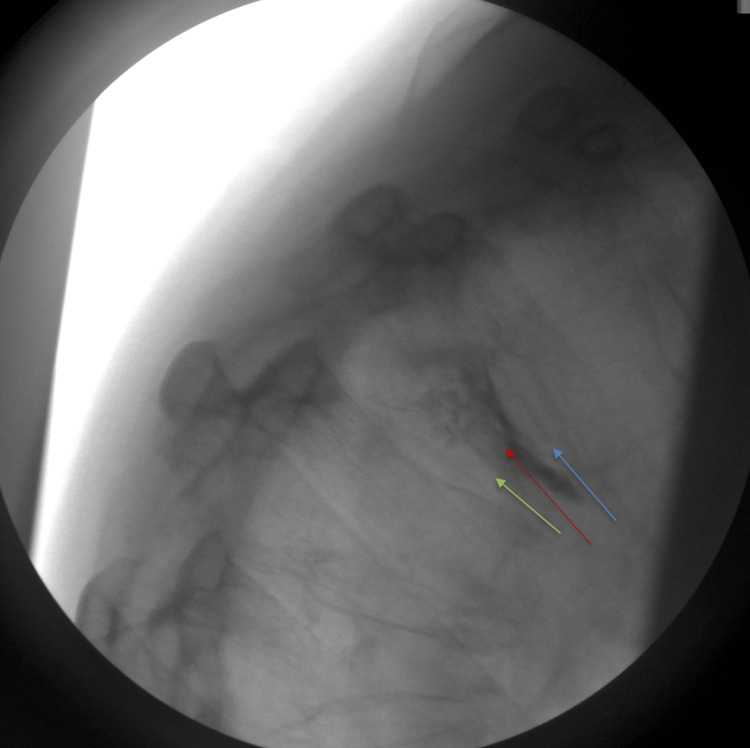
Postoperative kyphoplasty. Image demonstrating the restoration of vertebral bone height and stabilization from injected bone cement (red arrow). Superior and inferior endplates of the T5 vertebrae are indicated by the blue and green arrows, respectively

This curved needle successfully navigated around the concave, compressive anterior deformity to deploy the balloon and restore vertebral height. Bone cement was then injected to stabilize the vertebrae (Figure 6).

An intraoperative biopsy of the fractured bone was performed, which showed no evidence of neoplastic processes. Postoperative assessment indicated complete resolution of pain, and the patient has remained symptom-free during subsequent follow-up visits. A dual-energy X-ray absorptiometry scan revealed a T-score of -3.1, indicative of severe osteoporosis, necessitating further evaluation and management by the patient's primary care physician.

## Discussion

VCFs are a prevalent consequence of osteoporosis, particularly among postmenopausal women [8]. This demographic is frequently at risk due to diminished estrogen levels, as estrogen provides a protective effect against fractures by regulating bone remodeling [9]. When estrogen levels decline, bone density typically decreases, thereby increasing the risk of fractures [3,9]. VCFs are characterized by midline back pain, which often worsens with movement and is tender upon palpation [1,2,8]. While standard treatments for painful VCFs involve conservative approaches, minimally invasive procedures such as kyphoplasty are employed when conservative management fails [8]. Although this patient presented with a severe compression fracture secondary to her osteoporosis-induced Gibbus deformity, this case underscores the importance of minimizing risk as much as possible to significantly improve the patient's quality of life.

This case presented an anatomical challenge due to the patient's fixed thoracic kyphotic deformity, secondary to an osteoporotic VCF. The extent and morphology of vertebral collapse considerably constrained the trajectory and spatial flexibility necessary for the safe and effective deployment of a balloon tamp using conventional instrumentation. To address these limitations, a curved needle was introduced via a unipedicular approach. Curved instruments afford greater directional control within the vertebral body and can navigate convoluted and/or distorted fracture planes effectively [10,11]. This methodology enabled adequate vertebral filling and stabilization throughout the entire volume of the vertebral body [10,11]. Such an approach not only minimizes operative risk by reducing disruption to the vertebral structure but also demonstrates that, even in cases of complex deformities, this technique remains both feasible and efficient [11].

Although current practices favor the utilization of two trocars within a bipedicular approach, a meta-analysis conducted by Zhang et al. determined that the unilateral method demonstrates a more pronounced impact on enhancing post-kyphotic angles [12]. It also necessitates less operative time and a reduced volume of cement injection, thereby suggesting that the unilateral approach might be safer for elderly patients, who are more vulnerable and susceptible to underlying disease processes [11]. Furthermore, a comparison between percutaneous curved vertebroplasty and bilateral percutaneous kyphoplasty revealed that the former group experienced lower refracture rates, shorter operation times, and decreased fluoroscopy exposure (2.13% vs. 17.02%, respectively, p < 0.05) [13].

The successful application of a curved needle in this patient's case underscores the significance of versatile instrumentation options in spinal pathologies, where precision and gentle percussion are paramount to prevent harm. Furthermore, this supports existing literature advocating for the increased utilization of unipedicular approaches, which have demonstrated comparable, if not superior, efficacy relative to bipedicular access concerning pain alleviation and morbidity [13,14]. Specifically, this patient's complete pain resolution reflects literature showing that curved-needle augmentation provides pain outcomes comparable to bilateral straight techniques, while offering greater efficiency with shorter operative time, reduced cement use, fewer fluoroscopic exposures, and lower leakage risk [14]. Ultimately, it is essential to assess the patient's anatomy both before and during the procedure to identify the safest and most effective technique for treating VCFs.

## Conclusions

This case underscores the significance of a tailored approach in managing an anatomically complex VCF, particularly in cases of osteoporotic deformity secondary to the kyphotic angulation. Employing a unipedicular approach with a curved needle to access challenging fractures demonstrates how technical limitations can be effectively overcome, resulting in excellent clinical outcomes. Not only did the procedure reduce her visual analog scale (VAS) score from 10 to a sustained VAS score of 0, a 100% reduction in the patient's pain, at her two-week and eight-week follow-up, but it also facilitated the restoration of function with minimal trauma and adverse effects. The patient also reported being able to ambulate comfortably, sleep through the night, and perform her normal activities of daily living as done before the compression fracture. Postoperative imaging also confirmed correct placement of cement without migration or leakage from the vertebral body. This case highlights the need for continued advancements in kyphoplasty tools, procedures, and techniques to enhance patient outcomes, particularly among vulnerable patient populations.
